# Calcium Phosphate-Based Bioceramics in the Treatment of Osteosarcoma: Drug Delivery Composites and Magnetic Hyperthermia Agents

**DOI:** 10.3389/fmedt.2021.700266

**Published:** 2021-06-30

**Authors:** Tiê Menezes Oliveira, Fernanda Costa Brandão Berti, Sidney Carlos Gasoto, Bertoldo Schneider, Marco Augusto Stimamiglio, Lucas Freitas Berti

**Affiliations:** ^1^Department of Mechanical Engineering, Postgraduate Program in Biomedical Engineering, Federal University of Technology Paraná, Curitiba, Brazil; ^2^Department of Genetics, Federal University of Paraná, Postgraduate Program in Genetics, Curitiba, Brazil; ^3^Department of Mechanical Engineering, Postgraduate Program in Electrical Engineering and Industrial Informatics, Federal University of Technology Paraná, Curitiba, Brazil; ^4^Basic Biology of Stem Cells Laboratory, Carlos Chagas Institute-Fiocruz-Paraná, Curitiba, Brazil

**Keywords:** bioceramic material, calcium phosphate, hydroxyapatite, drug-delivery systems, magnetic hyperthermia, osteosarcoma

## Abstract

The use of biomaterials in medicine is not recent, and in the last few decades, the research and development of biocompatible materials had emerged. Hydroxyapatite (HAp), a calcium phosphate that constitutes a large part of the inorganic composition of human bones and teeth, has been used as an interesting bioceramic material. Among its applications, HAp has been used to carry antitumor drugs, such as doxorubicin, cisplatin, and gemcitabine. Such HAp-based composites have an essential role in anticancer drug delivery systems, including the treatment of osteosarcoma. In addition, the association of this bioceramic with magnetic nanoparticles (MNPs) has also been used as an effective agent of local magnetic hyperthermia. Further, the combined approach of the aforementioned techniques (HAp scaffolds combined with anti-tumor drugs and MNPs) is also an attractive therapeutical alternative. Considering the promising role of the use of bioceramics in modern medicine, we proposed this review, presenting an updated perspective on the use of HAp in the treatment of cancer, especially osteosarcoma. Finally, after giving the current progress in this field, we highlight the urgent need for efforts to provide a better understanding of their potential applications.

## Introduction

The emerging use of biomaterials in modern medicine has been constantly updated as new materials are developed. Several clinical applications for biomaterials include their use in the manufacturing of advanced surgical instruments, in plastic surgery, correction of anomalies, production of scaffolds (porous structures used as guide substrates for tissue regeneration) for regenerative medicine, in addition to their use as a structural function (1–3). Among the most commonly used biomaterials, polymeric, metallic, and ceramic materials stand out (4). Moreover, it is essential to highlight that for its application in clinical medicine, a particular biomaterial needs to have its biocompatibility guaranteed. According to D. F. Williams, biocompatibility is the ability of a material to perform with an appropriate host response in a specific application (5), making its use safe and suitable.

Among the ceramic biomaterials used in clinical practice, Hydroxyapatite (HAp) stands out. HAp is a mineral of the family of calcium phosphates, a natural constituent of bones and teeth, and widely used in the production of macroporous scaffolds (6–8). Among its main applications in medicine, we highlight HAp's use in bone grafts/implants (9, 10) for its osteointegration property, in addition to its use as a carrier of bioactive molecules, including drugs and growth factors (11–14). In this context, such bioceramics are used in a wide range of cases, from bone regeneration to cancer nanomedicine (15–17).

According to the National Cancer Institute (INCA) in Brazil, cancer is defined as a set of more than 100 diseases that have in common the disordered growth of cells, with the capacity to invade tissues and organs (18). It is currently the second leading cause of death globally, with an alarming increase in recent years (19). Among the different types of cancer, osteosarcoma—a malignant tumor that affects the connective tissue (20)—is highly prevalent in children and adolescents (0–19 years of age) (21, 22). In general, the usual treatment protocol for osteosarcoma is the resection of the primary tumor and bone metastasis associated with chemotherapy (23). The bone defect resulting from tumor resection is commonly filled with bone grafts in order to avoid fractures and/or deformations (24). Since there is significant complexity in removing tumor tissues, the resection procedure is usually followed by chemotherapy or radiation therapy (25, 26). However, the chemotherapy approach has the disadvantage of instantaneously release a large part of the anti-tumor drug right after its administration, reaching a peak of maximum concentration in the initial moments, followed by a rapid decline, which is unfavorable to treatment (17).

In this context, efforts have been made to obtain alternative treatment methods, with two research fronts being the most promising ones. The first is the use of HAp scaffolds as drug delivery composites. Associated with anti-tumor drugs, such as doxorubicin, cisplatin, and methotrexate (27–32), the gradual detachment of these molecules in specific places is guaranteed, also allowing the optimal *in loco* concentration of the respective drug, which is sustained for more extended periods, thus avoiding undesirable side effects (33–36). The second research front focuses on associating HAp with magnetic nanoparticles (MNPs), to guarantee their biocompatibility (37). The insertion of these nanoparticles in the body, followed by an increase in the local temperature—caused by the heating of the MNPs under the action of low-intensity external magnetic fields—is a method called local magnetic hyperthermia (MH). This technique can kill tumor cells, inducing apoptosis without damaging healthy tissues, since non-tumor cells tend to be more resistant to temperature variations due to the differential expression of specific proteins, including heat shock proteins (38, 39). Further, the magneto-mechanical stimulation of bone cells can favor bone proliferation, differentiation, and regeneration (40, 41). Also, the effect of the magnetic field alters the drug release kinetics in magnetic scaffolds (42), allowing a possible combined action. Collectively, we notice that different approaches have been demonstrated as promising and effective *in loco* strategies for bone tissue engineering, consequently for osteosarcoma therapy. Therefore, this review aims to provide an updated perspective about the use of HAp nanoparticles in the treatment of osteosarcoma, acting as drug delivery composites, and as auxiliary agents of MH, as well as in a combined approach, promoting bone repair and regeneration. Additionally, we conclude by discussing the urgent need for efforts in the emerging field of nanotechnology in bone tissue engineering and cancer therapy.

## Methods

We elaborated this review based on a critical bibliographic search through different databases, including the NCBI-PubMed, Medline, and Scientific Electronic Library Online (SciELO). We searched for articles written in English and published in the period between January 2015 and July 2020. This review included articles evaluating the use of calcium phosphate-based Bioceramics (both *in vivo* and *in vitro*) as an anti-tumor therapeutical approach, especially for the treatment of osteosarcoma.

## Results

### Basic Aspects of Calcium Phosphate-Based Bioceramics: Synthesis, Toxicity, and Scope in the Treatment of Osteosarcoma

Before revisiting the different approaches to the use of HAp nanoparticles in the treatment of osteosarcoma, it is important to mention relevant aspects about the synthesis of calcium phosphate-based bioceramics, as well as their toxicity and importance/limitation toward the treatment of osteosarcoma. Basically, there are different pathways to produce calcium phosphate-based bioceramics, including wet chemical precipitation, sol-gel synthesis, solid-state reactions, and even pyrolysis of polymeric precursors (43–46). Besides that, it is well-known that naturally found precursors as fishbone and eggshell can be used to produce HAp (46, 47), which could be considered an advantage. Concerning the calcium phosphate-based bioceramics' safety, several researchers have studied HAp nanoparticles and other phosphates' toxicity against healthy and cancerous cells, like Saos-2 (48–50). Results show little or no cytotoxicity against healthy osteoblasts. Moreover, HAp composite enhances osteoblast activity and reduces the expansion of cancerous cells (50). Once HAp is naturally found in hard tissue as the main component of the inorganic phase, low cytotoxicity is expected to synthetic HAp (51).

Additionally, since these bioceramics offer many significant properties for hard tissue regeneration, as well as other attractive properties [such as biocompatibility, biodegradation, non-toxicity, etc. (52)], their potential application in biomedical areas has rapidly expanded (53, 54). Today's technology allows the manufacturing of precisely designed scaffolds from bioceramics-based composites for specifics bone defects caused by resections, creating several possibilities and new opportunities for treatment. For instance, the use of HAp in different approaches for the treatment of osteosarcoma presents several advantages which are mentioned throughout the present manuscript (representing a potential alternative treatment with a focused and efficient action; combined with different strategies it also improves its desirable effects, etc.). As limitations of the use of HAp toward the treatment of osteosarcoma, as well as calcium phosphate-based bioceramics in general, we highlight the low number of studies investigating the anti-tumor/regenerative role of these bioceramics (both *in vitro*, and *in vivo*). We believe that this is the main limitation in this field at the present moment. In addition, is noteworthy the need for the technology to produce such pieces, since in the standard procedure—autologous graft—there is no need to do so (as the bone used to replace the removed tissue comes from the patient's body itself). In order to expand the research on the application of such bioceramics, it would be interesting to have access to new technologies to obtain such prototypes.

### HAp as Anti-tumor Drug Delivery Composites

**Figure 1:**
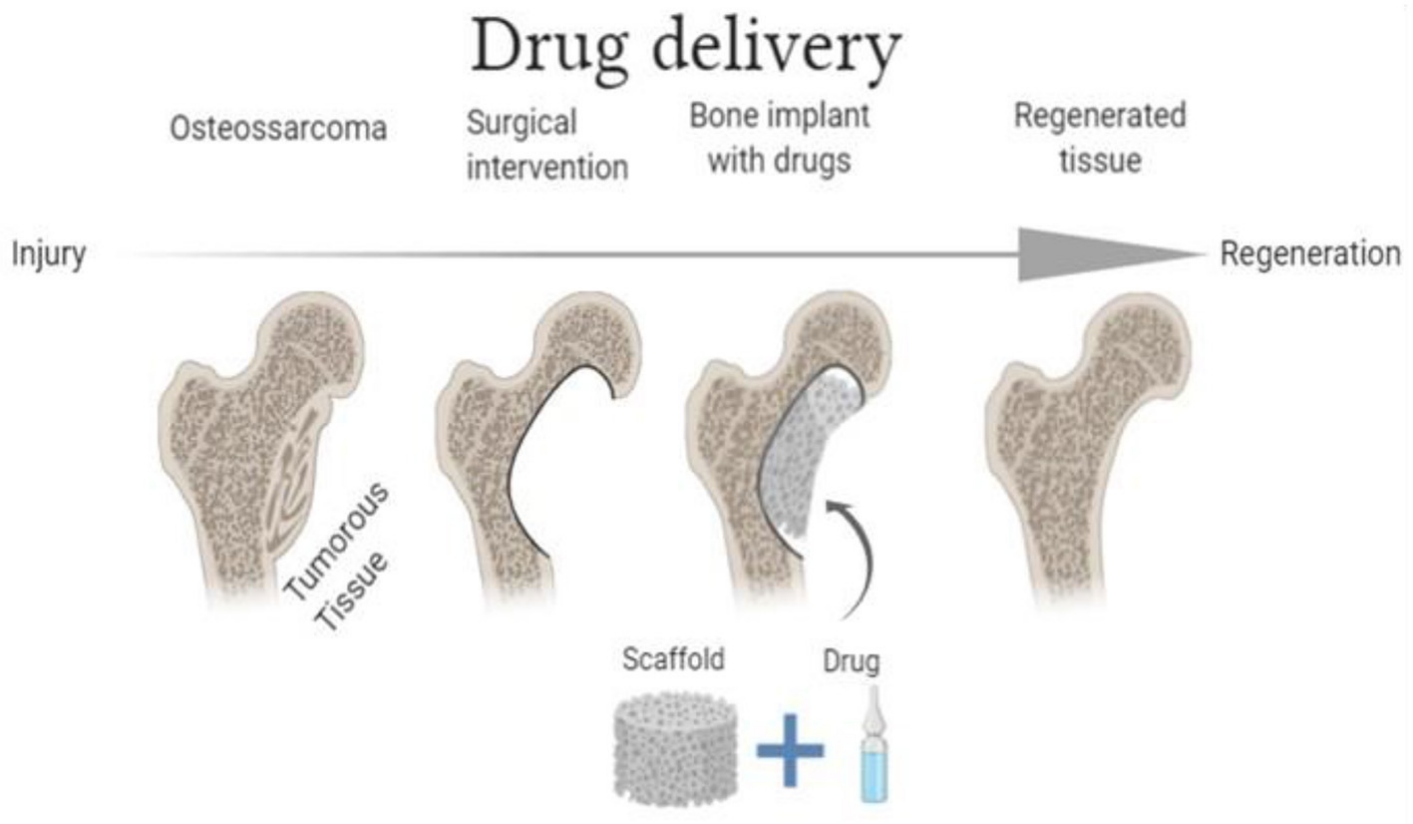
Schematic illustration of the functioning of drug delivery scaffolds (Image Source: created using BioRender).

Similar to other ceramic materials, HAp has some characteristics that limit its use in its pure form, including high hardness, low resistance to fractures, and fragility (55). To overcome these limitations, HAp-based composites and polymers such as gelatin, alginate, collagen, fibroin, chitosan, and cellulose are synthesized, aiming to produce biocompatible scaffolds presenting properties with those of natural biological tissues (56–60). Therefore, HAp biocompatible scaffolds can be used as anti-tumor drug delivery composites, carrying drugs such as doxorubicin. Such an anti-tumor agent is widely used in different chemotherapy therapeutical protocols, primarily due to its effectiveness against solid tumors. Despite that, doxorubicin's use is associated with several side effects (mainly cardiotoxic effects), especially related to its high dosage (61, 62). As an alternative, it has been proposed to administer this anti-tumor through scaffolds that allow its controlled and continuous release directly into the tumor microenvironment. Figure 1 schematically illustrates how anti-tumor drugs can be loaded in bioceramics-based scaffolds.

Rong et al. (63) analyzed various properties and applications of nanohydroxyapatite/collagen (ηHApC) scaffolds filled with Adriamycin (ADM) (trade name for doxorubicin), encapsulated in microspheres of poly(lactic-co-glycolic acid) (PLGA) (63). *In vitro* experiments demonstrated that such composite scaffolds provided a prolonged drug release for a period of up to 28 days. In addition, significant growth inhibition of human osteosarcoma lineage cells (MG-63) was observed, with higher ADM released concentration being associated with a superior cytotoxic effect. Further, *in vivo* experiments evaluated the immune response elicited by the subcutaneous implant of these scaffolds, confirming their remarkable biocompatibility. In rats, the implanted scaffolds did not generate a significant inflammatory response. Finally, to analyze the anti-tumor potential of these scaffolds *in vivo*, an osteosarcoma model in rats was designed through the hypodermal inoculation of MG-63 cells. Animals were divided into four groups. The first group did not receive any treatment (A), while the second one received an intraperitoneal injection of ADM (B). The third underwent a procedure where the scaffold loaded with PLGA/ADM microspheres was inserted into the tumor (C). Simultaneously, the fourth group received scaffolds without the microspheres containing the drug (D). By the end of the experiment, the scaffold-treated group containing the PLGA/ADM microspheres showed less tumor progression than the other treatment groups, demonstrating its promising anti-tumor effect. Moreover, it is noteworthy that this group had fewer adverse effects than the group treated only with the drug in its conventional form.

Besides carrying a single drug, anti-tumor drug delivery composites may also encapsulate different substances. Emerging evidence suggests that nanotechnology-based combinational drug delivery therapies (i.e., using two or more different anti-tumor drugs) seem to be more effective than those using only one chemotherapeutic agent (64, 65). Prasad et al. (66) proposed the use of Hydroxyapatite-poly(vinyl alcohol) core-shell nanoparticles for dual delivery of two important anti-tumor drugs—methotrexate and gemcitabine—for bone cancer treatment (66). Using such an approach, adequate release kinetics of both chemotherapeutic agents was observed, which was adequately demonstrated by *in vitro* experiments (25 and 60% of methotrexate and gemcitabine, respectively, were released in the first 10 days). Additionally, the cytotoxicity evaluation experiments with MG-63 cells of these loaded scaffolds demonstrated satisfactory results, with dose-dependent cytotoxicity being observed.

Further, Hess et al. evaluated the anti-tumor effect of the co-carriage of doxorubicin and cisplatin using microspheres of beta-tricalcium phosphate in porous HAp scaffolds *in vitro* (67). Assuming that bone substitutes with controlled release of anti-tumor drugs approach are an interesting option “to combat” remaining cells derived from bone tumor resection, the authors proposed using this strategy. *In vitro* drug release analysis was assessed from microspheres loaded with the chemotherapeutic agents individually and simultaneously. A higher concentration of the released drugs was observed when the chemotherapeutic agents were loaded separately. Such effect was observed for both anti-tumor drugs, with doxorubicin presenting a more prolonged diffusion-controlled release rate for over 40 days compared to the co-delivery system. Additionally, the cytotoxicity of the proposed systems was evaluated using MG-63 cells. Despite presenting a lower release rate, the co-delivery system induced the highest cytotoxicity effect, suggesting that the co-loading of different chemotherapeutic agents in scaffolds is highly promising for osteosarcoma therapy due to the synergistic impacts over a long period of more than a month.

Moreover, in addition to acting as potential carriers, hybrid scaffolds containing HAp can also assist in bone regeneration after resection, promoting osteogenic differentiation of mesenchymal stem cells (68). Adenosine, a nucleoside that directly affects bone metabolism in its phosphorylated form, plays a major role in modulating stem cell differentiation into osteoblasts and osteoclasts (69). Zhou et al. (26) proposed microspheres with promising dual function (26), simultaneously presenting properties on drug delivery for anti-osteosarcoma treatment and showing a potential role in osteogenic differentiation and bone regeneration. *In vitro* toxicity tests were performed with six osteosarcoma cell lines (including the MG-63 line) and *in vivo* (osteosarcoma animal model using the 143B line). The obtained results highly support the therapeutic efficiency of the evaluated approach and the scaffold's ability to promote osteogenic differentiation of human bone mesenchymal stem cells (hBMSCs) by activating the Adenylate-activated protein kinase (AMPK) signaling pathway. This pathway was confirmed through different techniques [such as cellular alkaline phosphatase staining, alizarin red staining, reverse transcription-quantitative PCR (polymerase chain reaction), and Western Blotting].

Therefore, several strategies based on the use of bioceramics as an anti-tumor therapeutical approach for the treatment of osteosarcoma have been proposed. Most recently, other studies have been carried out to evaluate HAp's ability to act as an anti-tumor drug delivery agent in osteosarcoma as well as in different cancers (70–72).

### HAp With Agents for Magnetic Hyperthermia

Currently, two different strategies based on nanoparticle-mediated hyperthermia stand out—treatments using ultrasound (73) or lasers (74)–, in order to generate heat. As for ultrasound therapy, it is essential to highlight that the variation in the sound speed somehow impairs the present procedure through the tissues (75). Similarly, the tissular structure attenuates the range of optical lasers (76). Therefore, the combined use of HAp scaffolds associated with MNPs appears as a promising method, especially for presenting fewer side effects and the ability to reach and act at different depths in the tumor (77, 78).

**Figure 2:**
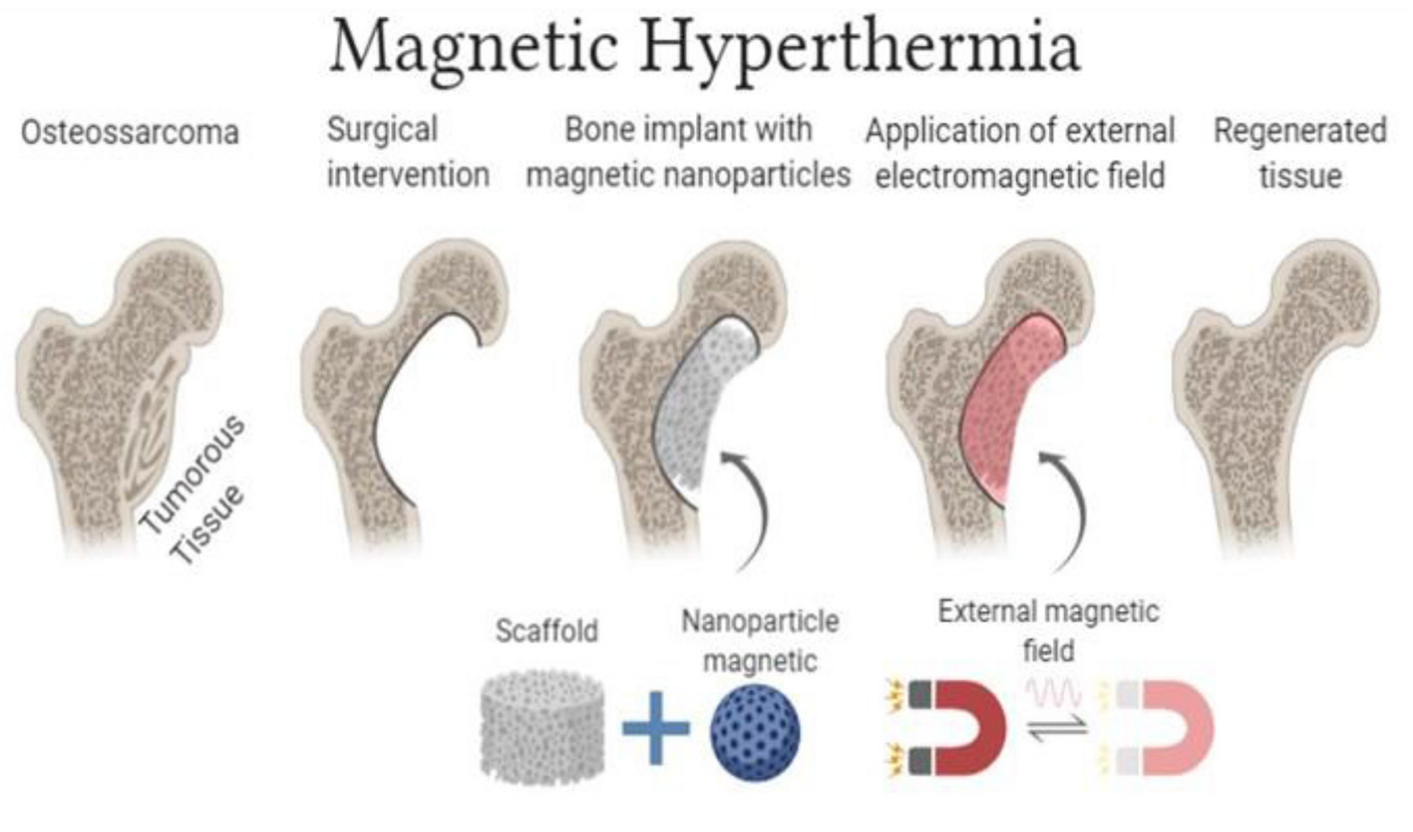
Schematic illustration of the functioning of MH scaffolds (Image Source: created using BioRender).

In such a context, HAp with MNPs is one of the new cancer treatment techniques approved for clinical tests, promoting better control of the energy supplied to the tumoral site (79). Figure 2 illustrates the use of scaffolds with MNPs. Among the desirable characteristics of MNPs, superparamagnetism stands out. Such property is defined as the material's ability to become highly magnetized when exposed to low-intensity magnetic fields, without residual magnetization after field removal (80). Magnetic hydroxyapatite nanoparticles (mHAp) are effective MH agents, especially due to their superparamagnetic properties associated with the excellent biocompatibility of this phosphate (81, 82). In 2018, Yang et al., synthesized HAp magnetic nanoparticles via co-precipitation with FeCl2 in order to evaluate the effect of hyperthermia *in vitro*, using a liver cancer cell line (HepG2) (83). Tumor cells were cultured with mHAps and exposed to an alternating magnetic field for 30 min, reaching temperatures up to 43 ± 0.5°C. Cell viability analyses indicated a considerable reduction in the experimental group (exposed to the induced hyperthermia) compared to controls (without exposure).

In addition to modifying HAp, aiming to guarantee magnetic properties to the particles, an alternative option is using this bioceramic to coat magnetic particles to synthesize MNPs with desirable biocompatibility. Mondal et al. (84) produced iron oxide (Fe_2_O_3_) nanoparticles coated with HAp (IOHAps) in order to assess their potential use as a promising nanomaterial for MH cancer treatment (84). In experiments using osteosarcoma cell lines (MG-63), IOHAps are biocompatible and capable of eliminating cytotoxicity induced by iron oxide. On the other hand, IOHAp could produce hyperthermia, leading to a mortality rate of MG-63 cells close to 100% within 30 min of exposure, with temperatures reaching ~45°C. Further, without the magnetic field, the synthesized nanoparticles show minimal or no cytotoxic effect on cell lines.

Moreover, Ereath Beeran et al. (85) synthesized a system comprising iron oxide particles coated with HAp to act as agents of local MH, as well as to be used in magnetic resonance imaging (MRI) contrast enhancement (85). The *in vitro* effectiveness of the system was evaluated by exposing HeLa cells (cellular lineage commonly used in studies to assess cell proliferation, cytotoxicity and mortality) to several concentrations of particles under magnetic fields of different intensities. Under some conditions (particle concentration of 2 mg/mL, the field of 33.8 mT, for 30 min), the iron oxide embedded hydroxyapatite super-paramagnetic particles were able to induce cell death by apoptosis at a rate of ~70%.

Additionally, Li et al. (86) published complete research in which they evaluated the osteogenic effects of HAp scaffolds modified with MNPs for the reconstruction of the bone defect after bone tumor resection (86). *In vitro* assays investigated the scaffold's anti-tumor potential by co-culturing them with MG-63 cells with subsequent exposure to alternating magnetic fields to achieve hyperthermia. Cellular adhesion and proliferation potential of the osteoblastic cell line MC3T3-E1 were also evaluated in order to determine the scaffold's osteogenic potential. Afterward, the scaffolds were implanted in bone defects artificially induced *in vivo* (in rabbits). Two control groups were included: one consisting of rabbits that did not receive any type of treatment, and another in which the animals were implanted with scaffolds without magnetic particles. Approximately 78% of MG-63 cells' mortality was achieved through MH and good cell adhesion, which was observed for scaffolds with and without MNPs. Interestingly, the *in vivo* bone defect repair experiment revealed that the composite scaffolds with MNPs had a good osteogenic capacity, with bone defects artificially induced being fully “recovered” within the twelfth week of treatment. No statistical differences were observed between the groups receiving scaffolds with and without MNPs.

Further, Adamiano et al. (87) investigated the potential use of magnetic calcium phosphates nanocomposites for the intracellular hyperthermia of different cancers, including bone and brain (87). Two superparamagnetic calcium phosphates were synthesized—iron-doped hydroxyapatite (FeHA) and iron oxide nanoparticles coated with amorphous calcium phosphate (Mag@CaP)—and tested to determine their anti-tumor effect over different cell lines, including osteosarcoma cells (K7M2-pCl). Hyperthermia experiments demonstrated that allowing cancer cells to uptake FeHA or Mag@CaP particles before employing an alternate magnetic field reduces cancer cell viability significantly more than running the same experiment on cells in superficial contact with the nanoparticles. The uptake of the particles was confirmed using immunofluorescent staining and flow cytometry, which showed that 44.9 ± 12.0% of the cells incubated with FeHA nanoparticles and 17.7 ± 1.6% of cells incubated with Mag@CaP displayed increased granularity due to uptake. While the effect was statistically highly significant for Mag@CaP or FeHA particles, it was negligible for Mag, highlighting the benefit of combining iron oxide with calcium phosphates. The anti-tumor properties of these composites were also evaluated in human glioblastoma cells and against primary lung fibroblasts, showing a similar effect as for osteosarcoma. Accordingly, the studies mentioned above highlight the promising anti-tumor effect of different HAp-based magnetic nanoparticles as agents of MH against tumors, especially osteosarcoma. As substantial and significant evidence increases, such strategies may become helpful and gain applicability shortly.

### Combined Approach

**Figure 3:**
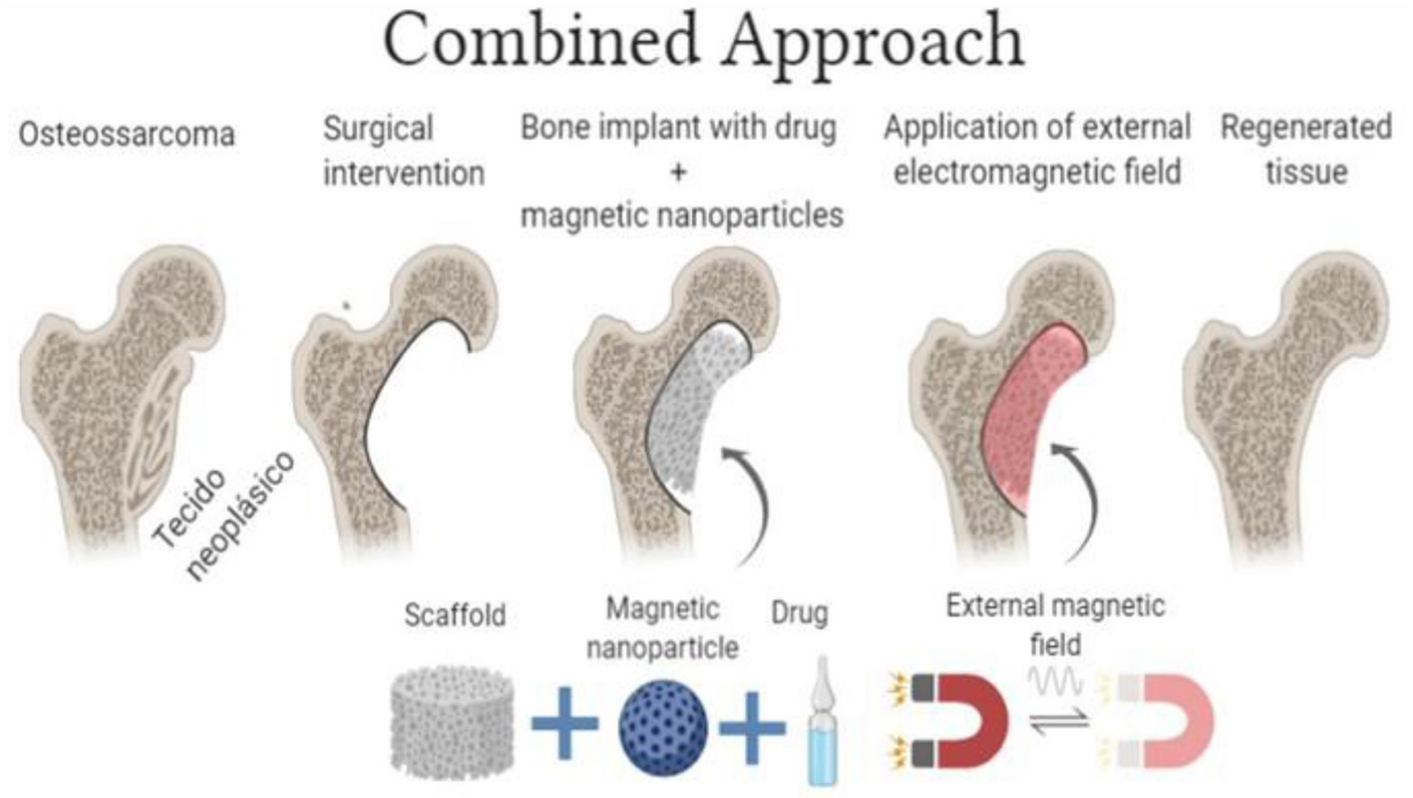
Schematic illustration of the combined approach (bioceramics scaffolds combined with an anti-tumor drugs) and MNPs (Image Source: created using BioRender).

In addition to the use of scaffolds loaded with drugs or MH agents individually, an alternative option is to combine these two strategies. Such a combined approach allows different results and final effects as the one observed independently. Magnetic fields acting on these scaffolds can induce the release of drugs through mechanical stimulation (vibration) and heating (88). Most recently, the possibility of “manipulating” magnetic nanoparticles through the application of external magnetic fields has gained much attention, mainly focusing on their combined use with drug delivery systems (37, 89, 90). The interest in MNPs' use in the synthesis of composites for scaffolds manufacturing for tissue engineering purposes had also increased (91, 92). Figure 3 presents an illustrative description of the combined approach.

Ficai et al. (42) evaluated the anti-tumor potential of a layer-by-layer scaffold produced from collagen, HAp, magnetite and cisplatin, with double action hyperthermia (induced by magnetite under the effect of an electromagnetic field) and sustained release of cisplatin in the treatment of bone cancer (42). Samples with one, three, five, and seven layers (LbL1, LbL3, LbL5, and LbL7, respectively) were obtained and tested. *In vitro* experiments evaluated the release kinetics of cisplatin, as well as its association with the respective number of layers in the scaffold. Results demonstrated that a more significant amount of layers reduces cisplatin bioavailability due to a slower diffusion (86.5 and 65% of cisplatin were released in the first 8 h for single and seven-layer scaffolds, respectively). Such behavior is mainly due to the reduction in the scaffold'*s* porosity. Further, a high mortality rate of HeLa tumor cells was induced by different analyzed samples, significantly inhibiting cell cycle progression (showing a dramatic decrease in the G2 phase as well as the appearance of a subG0 peak associated with apoptotic cell death) and confirming its anti-tumor activity. DNA replication (S phase) was also altered, probably as a consequence of cisplatin's mechanism of action. Different cytotoxicity levels were observed, with the cytotoxicity being dependent on the cisplatin content and the number of layers of the composite materials. Samples with a higher content of hydroxyapatite had more antitumoral activity because of their better absorption of cisplatin and, consequently, a higher concentration of cisplatin being present in the matrices.

Moreover, Iafisco et al. (88) investigated the interaction between the drug doxorubicin and iron-doped HAp superparamagnetic nanoparticles (FeHAp) in order to evaluate the kinetics of doxorubicin release *in vitro*, under conditions with and without the presence of an external magnetic field external (88). The amount of drug released from FeHAp composites under the magnetic field effect was higher than controls (pure HAp and FeHAp without the field action), mainly due to the magneto-mechanical agitation of the particles. *In vitro* experiments also demonstrated that FeHAp-associated doxorubicin exerted a cytotoxic role against a human osteosarcoma cellular lineage (Saos-2), similarly to the free administration of doxorubicin.

Most recently, after synthesizing scaffolds from composites consisting of calcium phosphates, collagen, and magnetic nanoparticles (Fe_2_O_3_ covered with chitosan), Cojocaru et al. (93) assessed the influence of radiation (X-rays) on these composites, simulating a post-surgical radiotherapy procedure (93). Moreover, the authors made the *in vitro* release evaluation of doxorubicin-loaded scaffolds and their interaction with osteosarcoma cells (MG63) through parameters as cytotoxicity. Preliminary results demonstrated that the X-rays were not capable of altering the composition and properties of the scaffolds. On the other hand, the gradual release kinetics of doxorubicin was observed, which was directly associated with a prolonged cytotoxic effect on osteosarcoma-derived cells. Therefore, suggesting the applicability and interesting final impact of a combined therapy against malignant bone tumors.

Taken all together, such results suggest that the combined approach not only produces a superior drug release but also modifies its release kinetics, indicating its promising application in the anti-tumor therapy of osteosarcoma.

## Discussion

Undoubtedly, cancer stands out among the pathologies of higher incidence, being one of the leading causes of death nowadays (94). Practical strategies for cancer management rely on several fronts, as the efficient screening and early diagnosis of cases, as well as the achievement of adequate and effective treatment. Scientists from different areas, using different approaches, continue investigating new anti-tumor treatment options to guarantee greater effectiveness and lesser side effects. Considering chemotherapy as the most widely used anti-tumor strategy and its systemic action—which may be a significant disadvantage (more side effects, also affecting healthy tissues)—it is vital to develop focused and targeted therapies.

Among the alternative approaches under investigation, the use of biomaterials stands out with promising applications in the treatment of malignant tumors, especially bone tumors, including osteosarcoma. In such a context, the use of bioceramics based on calcium phosphates is found either as drug delivery systems and/or as auxiliary agents of MH. Considering the ability of these composites to present a direct and “target” effect, which may represent an interesting and essential reduction of side effects when compared to other types of treatment, the use of biocompatible materials with anti-tumor properties has attracted the attention of several researchers.

**Figure 4:**
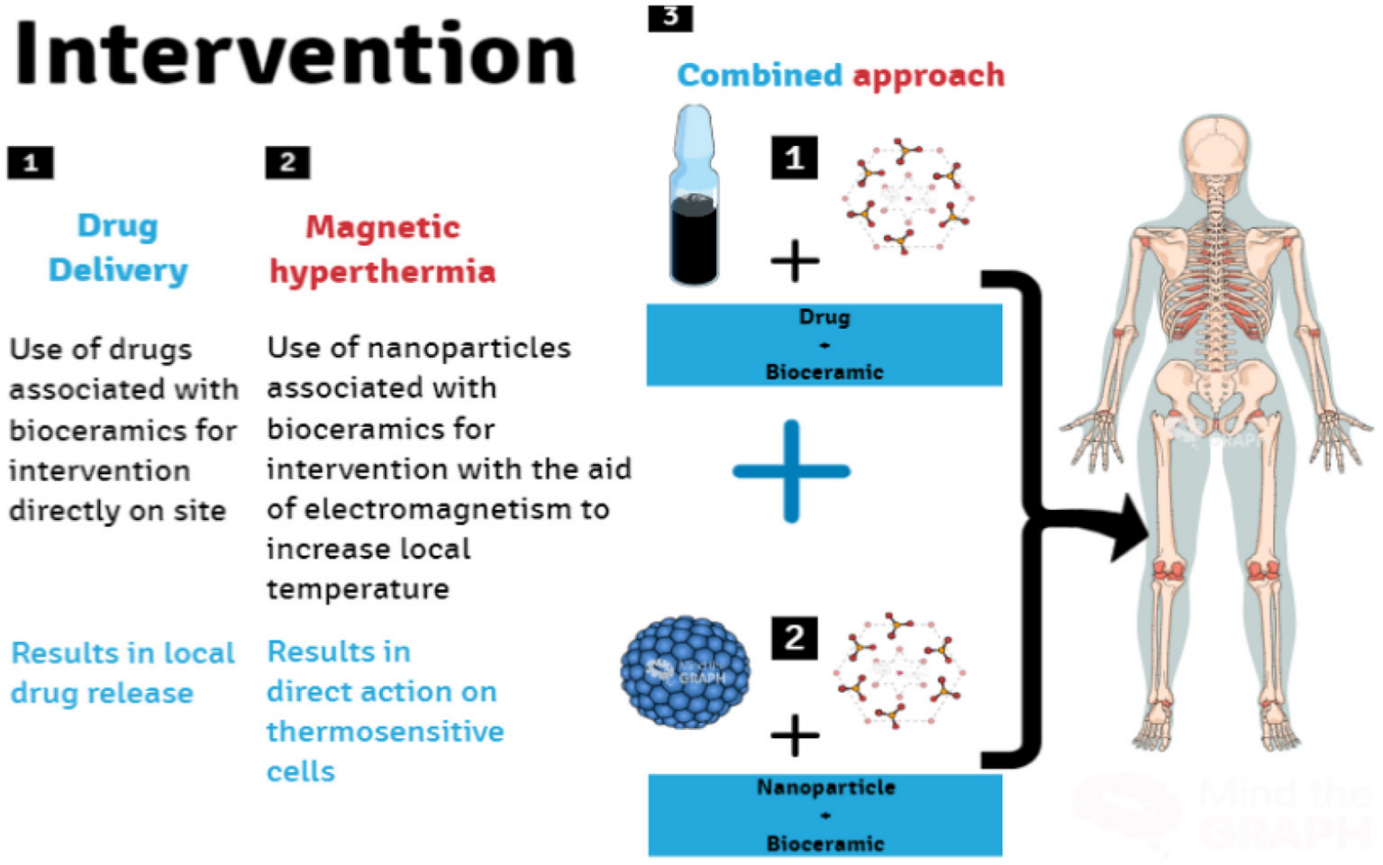
Research fronts for in loco intervention using bioceramics nanocomposites for osteosarcoma treatment. Bioceramics scaffolds as drug delivery systems for the anti-tumor drug(s) (1), scaffolds with MNPs (2), as well as scaffolds combined with the anti-tumor drug(s) and MNPs (3) (Image Source: created using Mind the Graph).

Among the different strategies using calcium phosphate-based bioceramics, the use of HAp as a drug delivery agent is included, with the main advantages of the local and non-systemic release of the associated drug, promoting a more punctual and efficient action. Another interesting strategy is the use of HAp as an auxiliary agent of MH, having the main advantage of the pronounced cytotoxic effect on tumor cells, associated with the local increase in temperature. In turn, in the combined approach, such desirable effects are added together. Additionally, a different release kinetics pattern is provided, which tends to be more prolonged, also accompanied by an increase in the concentration of the released drug. Figure 4 outlines the different approaches using bioceramics in the treatment of osteosarcoma.

Additionally, as future challenges, we highlight the immediate need for further studies in this field. We emphasize that despite all the efforts and results already achieved up to date, considering the research for bioceramics to be used in anti-tumor therapy for osteosarcoma (and other malignant tumors), there is still a low number of studies available. Although several studies have been carried out recently, mainly in the area of carriers of HAp-based medicines, the number of studies investigating the anti-tumor/regenerative role of these bioceramics *in vitro*, and especially *in vivo*, is still relatively low. See Table 1 for a better overview of references published until the present moment. Thus, it is crucial to evaluate these systems' behavior and their anti-tumor effects in more detail, including studies involving animals and carrying well-conducted and targeted clinical studies. Furthermore, the development of new technologies (equipment as well) to produce synthetic prototypes are also highly needed, as previously mentioned. Creating new composite materials and evaluating their potential biological effect are also extremely relevant. It is well-known that composite materials are produced from two or more different materials with notably distinct chemical and/or physical properties that, when merged, create a material with unique properties that are not depicted by any of the components in isolation (98). In such a context, the development of new bioceramics, as well as their combinations, may also create new opportunities yielding unpredicted outcomes.

**Table 1:** Summary of studies investigating different bioceramics-based strategies for the treatment of osteosarcoma.

**References**	**Details**	***In vitro***	***In vitro* assays**	***In vivo***	***In vivo* assays**	**Observed final effects**
**DDS-based studies**						
Yang et al. (95)	Rhein/PEG/nHAp conjugate spheres as DDS for DOX and Phosphorus-32 (32P)	✓	Release kinetics Bone affinity	✓	Biocompatibility (BALB/c nude mice) Tumor growth inhibition (BALB/c nude mice)	Sustained DOX release *in vitro* with pH dependence/ Enhanced bone affinity of nHAp by rhein modification/Bone-targeting ability of Rhein-PEG-nHAp contributed to the NP accumulation in the bone tumor *in vivo*
Prasad et al. (96)	Multilayer biodegradable core-shell NPs for co-delivery of MTX and DOX	✓	Release kinetics Cytotoxicity (MG-63)	X	-	pH-dependent release of drugs/ Concentration-dependent cytotoxicity in MG-63
Ghosh et al. (97)	DOX/HAp/PLGA NC	✓	Release kinetics Cell viability (MG-63) Cytotoxicity (MG-63)	X	-	pH-dependent drug release/ High controlled release of DOX was found in an acidic medium/ Desirable cytotoxicity of the NC toward MG-63 cells
Ram Prasad et al. (66)	HAp-poly(vinyl alcohol) core-shell NPs for dual delivery of MTX and GEM	✓	Release kinetics Cytotoxicity (MG-63)	X	-	25 and 60% of MTX and GEM, respectively, Released in the first 10 days/ Dose-dependent cytotoxicity
Hess et al. (67)	TCP beads incorporated in open-porous HAP matrix for co-delivery of CDDP and DOX	✓	Release kinetics Cytotoxicity (MG-63)	X	-	The Co-delivery system induced the higher cytotoxicity effect than individual delivery
Zhou et al. (26)	Microspheres filled with DOX (also as agents of osteogenic differentiation/ bone regeneration)	✓	Cytotoxicity (several cell lines, including MG-63)	✓	Cytotoxicity (BALB/c nude mice/ HOS-143B)	The hybrid biomaterial DDS exhibits an apparent therapeutic effect on osteosarcoma *in vitro/ vivo* and high performance in inducing osteogenic differentiation of hBMSCs
Rong et al. (63)	nHAp/collagen (nHApC) scaffolds filled with ADM	✓	Release Kinetics Cytotoxicity (MG-63) .	✓	Bone Repair (NZ rabbits) Biocompatibility (SD rats) Anti-tumor Potential (SD rats)	Prolonged drug release of up 28 days/ Good bone repair capacity/ Significant growth inhibition/ Biocompatible/ Significant anti-tumor effect
**MH-based studies**						
Ereath Beeran et al. (85)	Fe_2_O_3_ NPs coated with HAp for induced hyperthermia	✓	Cytotoxicity (HeLa)	X	-	Cell death by apoptosis at a rate of approximately 70%
Li et al. (30)	Evaluation of the osteogenic effects of HAp scaffolds modified with MNPs for the reconstruction of the bone defect after bone tumor resection	✓	Cytotoxicity (MG-63) Osteogenic potential (MC3T3-E)	✓	*In vivo* bone repair (NZ rabbits).	78% mortality of MG-63 cells were achieved through MH, as well as good cell adhesion/Scaffolds with MNPs presenting good osteogenic capacity, with bone defects artificially induced being fully “recovered” within the twelfth week of treatment
Adamiano et al. (87)	Magnetic NC for MH	✓	Cytotoxicity (several cell lines, including K7M2-pCl)	X	-	Intracellular NPs caused low cell viability on cancer cells under low power alternate magnetic fields
Yang et al. (83)	mHAps for induced hyperthermia	✓	Cytotoxicity (HepG2)	X	-	Reduction on cell viability (35% cell death after treatment with MH)
Mondal et al. (84)	Fe_2_O_3_ NPs coated with HAp for induced hyperthermia	✓	Cytotoxicity (MG-63)	X	-	Good biocompatibility, near 100% cell mortality within 30 minutes exposure, with temperatures reaching approximately 45°C
**Combined approach-based studies**						
Cojocaru et al. (93)	Investigation of the capacity of X-rays modifying the scaffolds (CaP/collagen/MNPs) properties.	✓	Release kinetics Cytotoxicity (MG-63)	X	-	X-rays were not capable of altering the composition and properties of the scaffolds/Gradual release kinetics of doxorubicin was directly associated with a prolonged cytotoxic effect on MG-63 cells
Iafisco et al. (88)	DOX loaded Iron-doped HAp NC for kinetics evaluation	✓	Release kinetics Cytotoxicity (Saos-2)	X	-	Higher amounts of drug released under magnetic field/Cytotoxicity similar to free administration of DOX
Ficai et al. (42)	Layered scaffold (Collagen/HAp/ magnetite/CDDP) as MH agent and DDS	✓	Release kinetics Cytotoxicity (HeLa)	X	-	Samples with a higher content of HAp had more antitumoral activity because of their better absorption of cisplatin and, consequently, a higher amount of cisplatin being present in the matrices

*Here, DDS-based, MH-based, and Combined Approach-based studies are presented in chronological order of publication (from the most recent to the oldest publication). ✓, performed; X, not performed; [ref], reference; A549, Human lung cancer cell line; BSA, Bovine serum albumine; CaP, Calcium phosphate; CDDP, cis-dichloro diammine-platinum or Cisplatin; DDS, Drug delivey system; DOX, Doxorubicin; FA, Folic acid; Fe_2_O_3_, iron oxide; GEM, Gemcitabine; HAp, Hydroxyapatite; hBMSCs, Human bone marrow stromal osteoprogenitor cells; HeLa, immortal cell line used in scientific research derived from cervical cancer cells; HepG2, human liver cancer cell line; HOS-143B, human osteosarcoma cell line (such cell line is generally subcutaneously injected in order to establish an in vivo osteosarcoma model); K7M2-pCl, Neo mouse osteosarcoma cell line; MC3T3-E, osteoblast precursor cell line derived from mouse calvaria; MDA-MB-231 eGFP, breast cancer cell line; MG-63, human osteosarcoma cell line; MH, Magnetic hyperthermia; mHAp, Magnetic hydroxyapatite; MNP, Magnetic nanoparticles; MTX, Methotrexate; NC, Nanocomposite; nHAp, Nanohydroxyapatite; NP, Nanoparticle; NZ, New Zealand; PC3, human prostate cancer cells; PLGA, poly(lactic-co-glycolic acid); PtPP, anti-tumor platinum complex; Saos-2, human osteosarcoma cell line; SD, Sprague–Dawley; Se, Selenium; Se$O_{3}^{2-}$, selenite; TCP, Tricalcium phosphate; WEHI-164, mouse fibrosarcoma cells*.

Gathering efforts to expand the range of different promising bioceramics/strategies for application in cancer therapy, better characterizing such materials to reproduce their expected anti-tumor effects in various studies, and assessing each strategy in a global and integrated way is crucial to make possible, in the near future, the use of some of these strategies in the clinical practice.

## Conclusion

The potential use of bioceramics in modern medicine is becoming more common each day, especially in treating different cancers. Due to its attractive properties, the use of HAp in the treatment of osteosarcoma has gained attention. Through various approaches, HAp may be used in such a context. First, HAp may be used as a drug delivery agent carrying anti-tumor drugs, promoting a more punctual and efficient action. Also, HAp may be associated with MNPs, acting as an auxiliary agent of MH.

Furthermore, another interesting option is to combine these two strategies, where desirable effects of both approaches are added together, representing a promising strategy to treat osteosarcoma. In this review, we present an updated perspective on using calcium phosphate-based bioceramics through such different approaches, highlighting important aspects of the different addressed strategies. Certainly, this is a research field under recent construction, and we highlight the urgent need for efforts to provide a better understanding of their potential applications, as well as to develop new strategies based on the unique properties that may emerge through the use of two or more materials to produce new bioceramics.

## Author Contributions

TO: conceptualization, methodology, writing—original draft, and writing—review & editing. FB: methodology, conceptualization visualization, writing—original draft, and writing—review & editing. SG: writing—original draft, visualization, and writing—review & editing. BS and MS: supervision, writing—original draft, and writing—review & editing. LB: conceptualization, methodology, supervision, visualization, writing—original draft, and writing—review & editing. All authors contributed to the article and approved the submitted version.

## Conflict of Interest

The authors declare that the research was conducted in the absence of any commercial or financial relationships that could be construed as a potential conflict of interest.

## Abbreviations

ADM: Adriamycin
AMPK: Adenylate-activated protein kinase
FeHA: iron-doped hydroxyapatite
HAp: Hydroxyapatite
hBMSCs: human bone mesenchymal stem cells
HeLa cells, cellular lineage commonly used in studies to assess cell proliferation: cytotoxicity and mortality
HepG2: liver cancer cell line (HepG2)
INCA: National Cancer Institute
IOHAp: iron oxide (Fe_2_O_3_) nanoparticles coated with HAp
K7M2-pCl: including osteosarcoma cells (K7M2-pCl)
LbL1, one layer—layer-by-layer scaffold produced from collagen, HAp: magnetite and cisplatin
LbL3, three-layer—layer-by-layer scaffold produced from collagen, HAp: magnetite and cisplatin
LbL5, five layer—layer-by-layer scaffold produced from collagen, HAp: magnetite and cisplatin
LbL7, seven-layer—layer-by-layer scaffold produced from collagen, HAp: magnetite and cisplatin
Mag@CaP: iron oxide nanoparticles coated with amorphous calcium phosphate
MC3T3-E1: Cellular adhesion and proliferation potential of the osteoblastic cell line MC3T3-E1
MG-63: human osteosarcoma lineage cells (MG-63)
MH: magnetic hyperthermia
MNPs: magnetic nanoparticles
MRI: magnetic resonance imaging
FeHApC: nanohydroxyapatite/collagen
PCR: polymerase chain reaction
PLGA: poly(lactic-co-glycolic acid)
Saos-2: human osteosarcoma cellular lineage (Saos-2).
